# The impact of motherless paternity testing in a South African population

**DOI:** 10.4314/ahs.v21i1.48

**Published:** 2021-03

**Authors:** André A De Kock, Jean JF Kloppers

**Affiliations:** 1 Department of Haematology and Cell Biology, School of Pathology, Faculty of Health Sciences, University of the Free State, Bloemfontein, South Africa; 2 Universitas Academic Unit, Department of Haematology and Cell Biology, National Health Laboratory Services, Bloemfontein, South Africa

**Keywords:** Motherless paternity testing, South African population

## Abstract

**Background:**

Paternity investigations play an important role in determining biological relatedness, and in South Africa, the outcome of these investigations impacts medical, judicial and home affairs decisions. Short Tandem Repeat (STR) analysis is utilised to perform paternity and kinship analysis, due to the polymorphic nature of STR loci. The cost associated with paternity testing is high, and there is a demand for motherless testing.

**Objectives:**

This study aims to determine what the impact of motherless testing would have been by evaluating 6182 paternity trio cases.

**Methods:**

The AmpFLSTR™ Identifiler™ PCR Amplification kit was used to profile each of the trio cases. A scenario was created where the mother was eliminated from the test results to determine if the paternity outcome would change.

**Results:**

Putative fathers were excluded in 27% of all cases, and in 2.5% of those cases, putative fathers would have been falsely included, had the mother not been tested. These false inclusions are attributed to coincidental STR loci that are shared between the mother and the putative father. The addition of loci to the STR profiling kit may resolve the issue; however, comparable STR data with more loci will have to be evaluated to ensure it overcomes the issue of coincidentally shared loci between unrelated individuals.

**Conclusion:**

We would recommend that within our setup and within similar setups, the mother always be included for testing, except in extreme scenarios such as death. False inclusion of putative fathers could have serious legal implications for testing laboratories.

## Introduction

Paternity and kinship investigations play a central role in determining biological relatedness.^1^ These investigations have become much more than a personal interest in family relations, considering that the outcome of these investigations is important in medical, judicial and home affairs cases.^2^–^3^ Worldwide, DNA Short tandem repeats (STR) analysis is used for paternity and kinship testing, as well as forensics.^4^ STR refers to short, tandemly repeated sequences of between 2 to 6 base pairs in length. STR loci are highly polymorphic and are found all over the eukaryotic genome. STR loci are polymorphic because the number of tandem repeats at specific STR loci varies between individuals, making these loci optimal for human identification purposes.^4^

The consensus in paternity testing is that when more than two STR loci mismatches occur between the child and the putative father, paternity is most often excluded. 5–6 Putative fathers are not excluded on one or two STR loci mismatches, as STR mutations can occur as a result of polymerase template slippage during the STR amplification process.^5^ When in doubt, the paternity index should be calculated, as it indicates the probability of paternity between the tested individuals.^7^ Preferably, a paternity test consists of a trio of individuals, including the mother, the child and the putative father. Our paternity testing facility has seen a ten-fold increase in cases over the past five years. Increased cases are specifically linked to the South African Department of Home Affairs, to assist in citizenship cases, as well as birth registrations. Consequently, there has been a demand for motherless paternity testing to be conducted, considering the cost implication of these tests. In some instances, the mothers are also not available for testing due to various reasons, including that the mother is deceased, or her geographical location is unknown at the time of the investigation. Motherless paternity testing can, however, lead to false paternity inclusions, due to coincidental STR loci matches between two unrelated pairs.^8^ The cases that are investigated usually presents with unfamiliar family backgrounds, as many individuals can't positively identify specific biological relationships. An example of such a case is where the child might identify an uncle as a putative father. Considering that the uncle and putative father may have very similar STR profiles, it can lead to false inclusion of paternity. In a study containing 5253 pair-wise combinations of STR loci, 3.71% of pair-wise combinations occurred in unrelated individuals. These findings confirm the probability of coincidental matches between two unrelated individuals that could lead to false inclusion of paternity. The power of exclusion for paternity in duo cases (child and putative father) was found to be inadequate, with special reference to the Identifiler™ STR-kit that we also utilise in our laboratory for paternity testing.^9^ The risk of falsely including a father in duo paternity testing, has been confirmed in several studies.^10^–^12^ The current policy at our paternity testing facility is to always include the mother in the paternity investigation, with exception to cases where the mother is demised or has abandoned the child.

## Aim

There has been an increased demand at our facility to perform motherless paternity testing in cases where the mother is available for testing. Therefore, this study aimed to investigate what the effect of motherless paternity testing would be on the outcome of paternity in excluded trio cases.

## Methods

### Population group

A retrospective data analysis study was performed, including 6182 trio paternity cases that were done at our facility between 2003 and 2018. The STR profiles of each individual were determined using the AmpFL-STR™ Identifiler™ PCR Amplification kit (Thermo Fischer Scientific, ZA). Cases, where the mother was not tested, were excluded from the study and in the case of more than one child involved in the investigation; each additional child was included as a separate trio paternity case. Ethics approval for the study was obtained from the Health Sciences Research Ethics Committee at the University of the Free State in Bloemfontein, South Africa (UFS-HSD2019/0545/2805).

### Data collection procedure

The trio paternity cases were investigated, and the total number of exclusions were counted. The excluded trio cases were then subjected to a scenario where the mother in each case was eliminated from the investigation. The STR profiles of the child and the putative father were evaluated to then determine if the outcome of the paternity results would have been different if the mother was not tested. The probability of paternity was calculated in duo cases where the outcome of the paternity changed from an exclusion to an inclusion, using locally available STR loci frequencies. A probability of paternity (%) above 99% was deemed as an inclusion.^2^ A case presentation, depicting the STR profiles and the outcome of the paternity test with and without the mother included in the test is used to illustrate the impact of motherless paternity testing.

In addition, the STR profile data collected was also used to determine the total number of STR loci putative fathers were excluded on. Also, the percentage of cases where a difference was observed in the total number of STR loci excluded on without the mother being included in the test was investigated.

### Data analyses

The collected data was imported into Microsoft Excel® (2016), which was subsequently used for data calculations and graph designs. Results are expressed as percentage values, and bar graphs are used to depict the STR loci data.

## Results

**Figure 1:**
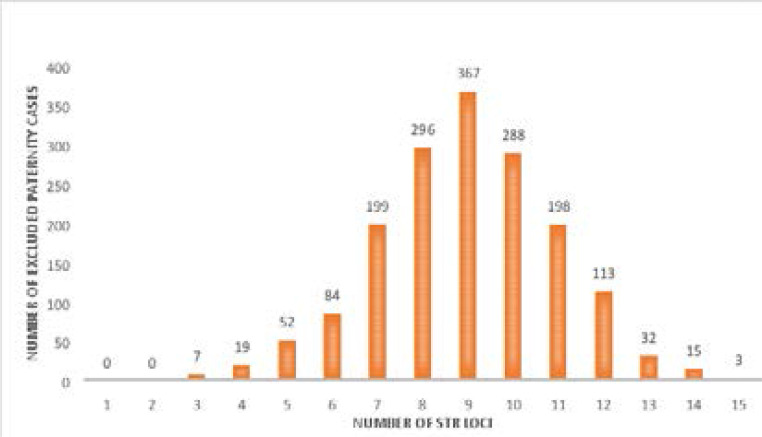
A bar graph depicting the number of Short Tandem Repeat loci in excluded paternity cases when the mother was included in the paternity test.

In total, 27% (n=1673/6182) of tested putative fathers were excluded from paternity. The total number of STR loci ranged between 3 and 15 loci in the excluded cases when the mother was included in the investigation (Figure 1).

In 89.3% (n=1494/1673) of the excluded cases, the total number of STR loci putative fathers were excluded on changed when the mother was not included in the investigation. Only in 179 (10.7%) investigated cases were there no difference in the number of excluded STR loci, whether the mother was included or not.

In total, 2.5% (n=42/1673) of the total excluded cases would have had a different outcome to the paternity results, if the mother was not tested. The probability for paternity in all the cases that changed to an inclusion was calculated to be above 99.98%. In tables 1 and 2 below paternity results are depicted for the 16 STR loci when the mother is also tested (table 1), and the paternity results for the same case when only the child and the putative father are tested (table 2). Consequently, these tables are an example that depicts a spurious paternal inclusion due to motherless testing being performed. In table 1, the putative father was excluded on 7 of the STR loci, with a 0% probability of paternity. In contrary, when the mother was not tested (table 2), the putative father is falsely included with no exclusion on any of the STR loci with a probability of paternity of 99.9%.

**Table 1:** Example of a paternal exclusion involving a mother, child and a putative father

STR locus	Mother	Child	Putative Father	Excluded (Yes/No)
**Amelogenin**	X/X	X/X	X/Y	
**D8S1179**	15/15	13/15	12/15	Yes
**D21S11**	29/32.2	31.2/32.2	28/31.2	No
**D7S820**	9/10	10/10	10/10	No
**CSF1PO**	11/12	11/12	11/13	No
**D3S1358**	15/16	16/16	16/17	No
**THO1**	9/9	7/9	7/7	No
**D13S317**	11/12	12/12	11/12	No
**D16S539**	10/12	10/11	9/10	Yes
**D2S1338**	21/21	21/25	19/21	Yes
**D19S433**	13.2/14	13/13.2	13/13	No
**vWA**	16/16	16/17	16/17	No
**TPOX**	9/11	9/10	9/11	Yes
**D18S51**	17/19	15/17	17/20	Yes
**D5S8181**	8/12	11/12	12/12	Yes
**FGA**	23/23	23/27	23/25	Yes

*STR: Short Tandem Repeat

**Table 2:** Example of a spurious paternal inclusion due to motherless testing

STR locus	Child	Putative Father	Excluded (Yes/No)
**Amelogenin**	X/X	X/Y	
**D8S1179**	13/15	12/15	No
**D21S11**	31.2/32.2	28/31.2	No
**D7S820**	10/10	10/10	No
**CSF1PO**	11/12	11/13	No
**D3S1358**	16/16	16/17	No
**THO1**	7/9	7/7	No
**D13S317**	12/12	11/12	No
**D16S539**	10/11	9/10	No
**D2S1338**	21/25	19/21	No
**D19S433**	13/13.2	13/13	No
**vWA**	16/17	16/17	No
**TPOX**	9/10	9/11	No
**D18S51**	15/17	17/20	No
**D5S8181**	11/12	12/12	No
**FGA**	23/27	23/25	No

*STR: Short Tandem Repeat

## Discussion

The number of paternity cases increased ten-fold over the past five years at our testing facility. The increase in testing volumes is due to late birth registrations and immigration cases from the South African Department of Home Affairs that require putative fathers to prove paternity. Paternity investigations have a tremendous cost implication on the parties involved, and consequently, there has been requests for motherless testing to be performed. The investigated cases in the study was aimed to determine what the outcome of motherless testing would have had on the paternity results. In order to evaluate what the effect of motherless testing would be, a scenario had to be created where the mother's STR profile was eliminated from the paternity investigation in the excluded cases. Consequently, the difference in the number of STR loci on which putative fathers were excluded could then be compared with and without the mother being tested, as the number of excluded loci subsequently determines the outcome of the paternity result.

The total number of STR loci on which fathers were excluded, ranged between 3 and 15 loci, with the majority of cases ranging between 7 and 11 loci. There was an 89.3% (n=1494/1673) change in the total number of STR loci on which putative fathers were excluded on when the mother was not tested. Only 179 (10.7%) of the excluded cases had no change in the total number of excluded STR loci, irrespective of whether the mother was tested. In essence, the difference in the total number of STR loci putative fathers was excluded on when the mother was not tested, indicated that motherless testing would have an impact on the final outcome of the paternity result.

Consequently, in 2.5% (n=42/1673) of all excluded cases investigated, the putative father would have been falsely included, due to coincidental STR loci shared between the mother and the putative father. In the study conducted by De Ungria et al. (2002)^8^, pair-wise combinations of STR loci occurred in 3.71% (n=195/5253) of unrelated individuals, and consequently, the current study is comparable with their findings. The paternity results depicted in table 1 and 2 is further support for the inclusion of the mother in testing. The putative father was excluded based on 7 STR loci, with a 0% probability of paternity in the investigation where the mother was included (table 1). On the contrary, when the mother is not tested, the putative father is included with at least one STR loci match per locus (table 2), evident from a paternity probability calculated to be 99.99%.

## Conclusion

Paternity testing is often being utilised in South Africa, especially referring to cases where positive identification of individuals is required in the medical, judicial and home affairs departments. In our setup, parties involved in testing is responsible for the cost, hence the increased demand for motherless testing. The outcome of this study does not support motherless paternity testing, as cases of false inclusion can occur if the mother is not tested. The use of STR profiling kits with more STR loci may potentially resolve the issue of coincidental loci between individuals, however, comparable data from these kits will have to be evaluated in order to determine if the issue of false inclusion can completely be resolved within our population. We would thus recommend that for the time being, that facilities that utilise the 16 STR loci kits always include the mother, with exceptions to the rule in extreme cases such as the mother being deceased or in cases of child abandonment. In such instances, paternity reports should clearly indicate that should the mother have been included in the test; a different result could be obtained. Motherless paternity testing could have dire financial and legal consequences, not only for the testing facility, but also for the wrongly included fathers, and should therefore only be considered in exceptional circumstances.
